# Healthcare professionals' challenges and solutions in providing palliative care to patients with severe chronic obstructive pulmonary disease

**DOI:** 10.7189/jogh.16.03004

**Published:** 2026-02-20

**Authors:** Sylvia McCarthy, Jayakayatri Jeevajothi Nathan, Ee Ming Khoo, Zee Nee Lim, Nik Sherina Hanafi, Norita Hussein, RESPIRE Collaboration

**Affiliations:** 1 Hospis Malaysia, Kuala Lumpur, Malaysia; 2 Department of Primary Care Medicine, Faculty of Medicine, Universiti Malaya, Kuala Lumpur, Malaysia

## Abstract

Palliative care significantly improves the quality of life for individuals with advanced chronic diseases. However, access in Malaysia remains limited, particularly for patients with non-malignant conditions such as chronic obstructive pulmonary disease (COPD). Here, we discuss the barriers and solutions identified through a nine hour in-person stakeholder workshop to co-develop practical initiatives for integrating palliative care into the management of severe COPD. Key challenges in the assessment of severe COPD included limited training in holistic care, resource constraints, and fragmented care pathways. In management, issues included low awareness and availability of pulmonary rehabilitation, uncertainty in initiating palliative care and difficulty accessing opioids, limited provider training, and patient-level barriers such as stigma and low health literacy. Proposed solutions included cascade training, decentralised care models, strengthened communication skills, and integrated care pathways. This workshop highlighted the need for multidisciplinary training, system-level integration, and culturally responsive care models to improve palliative access for patients with severe COPD. It also underscored the importance of policy engagement to address structural barriers such as opioid regulation and fragmented care. This approach offers a promising model for capacity building in other resource-limited settings.

Chronic obstructive pulmonary disease (COPD) is a prevalent chronic respiratory condition affecting approximately 11.7% of the global population aged 30–79 years [1]. It is the third leading cause of mortality worldwide, responsible for around 6% of all deaths, and ranked 7th among the World Health Organization’s top 10 causes of disability-adjusted life years in 2019 [2,3]. In Malaysia, COPD affects an estimated 4.7% of the adult population, with 4.6% of these individuals experiencing moderate to severe manifestations of the disease [4,5].

Severe COPD is associated with a high symptom burden, persistent breathlessness, fatigue, anxiety, depression, and frequent exacerbations that often lead to emergency admissions. Despite its progressive and life-limiting nature, the palliative care needs of these patients are frequently overlooked. Many individuals succumb to their illness without ever receiving palliative support [6]. Unlike cancer, COPD has a slow and unpredictable trajectory, making it difficult to determine when to initiate palliative care and end-of-life discussions [7,8]. A qualitative study involving patients and their healthcare professionals (HCPs) with GOLD stage D COPD revealed multiple unmet needs [9], such as limited understanding of disease prognosis, poor access to holistic and coordinated care, and suboptimal management of physical and psychosocial symptoms. Cultural norms, religious beliefs, and hierarchical healthcare structures further shaped patient communication and access to services. To address these issues, we convened a multidisciplinary workshop with HCPs to explore barriers and facilitators in providing palliative care for patients with severe COPD, and to identify practical, contextually relevant solutions.

A nine-hour in-person workshop was conducted with 25 purposively selected participants representing a broad spectrum of clinical disciplines routinely involved in the care of patients with severe COPD. Participants were identified through collaboration with government-funded health facilities and institutions, including public primary care clinics, university hospitals, and other public sector bodies, to ensure representation from across the public health care system. The group included professionals from primary care medicine (n = 16), respiratory medicine (n = 1), palliative medicine (n = 1), rehabilitation medicine (n = 3), physiotherapy (n = 3), and internal medicine (n = 1). Participants held various roles, including professors, specialists, rehabilitation officers and medical officers. Of the 25 participants, 22 were female and 3 were male. The majority had over 10–15 years of clinical experience.

The workshop was informed by the findings from the aforementioned qualitative study [9]. Participants were divided into four multidisciplinary groups of six to seven members each. Two structured discussion sessions were held, guided by a semi-structured discussion guide. The first session focused on identifying barriers and solutions in conducting holistic assessments, including physical, psychological, social, and spiritual dimensions. The second session explored challenges and strategies related to the management of severe COPD in palliative care, including continuity of care, symptom control, and advance care planning. Each group presented a summary of its discussion during a plenary session, followed by a facilitated synthesis of shared challenges and proposed solutions across groups. While no formal consensus techniques were applied, the discussions were consolidated in real time, with terms such as ‘widely discussed’ or ‘commonly raised’ used to reflect the prominence of each issue.

## OBSERVATIONS

### Multidisciplinary and integrated care: gaps and opportunities

Participants strongly endorsed the value of multidisciplinary collaboration in addressing the unmet needs of patients with severe COPD. However, they also highlighted several challenges, including the limited availability and involvement of palliative care specialists in managing patients with severe COPD, inconsistent referral pathways, and fragmented care delivery across health settings. These findings reflect broader international evidence demonstrating the benefits of integrated care pathways in high-income countries such as the UK and Australia, where primary care, respiratory, and palliative teams work in tandem [10–12]. Implementing such models in Malaysia remains challenging due to evolving infrastructure, limited institutional support, and underdeveloped referral mechanisms [13].

### Holistic assessment: training, resources, and system barriers

Training-related challenges were widely discussed in the workshop. Participants cited a lack of skilled personnel, low confidence in addressing psychosocial or spiritual issues, and a general tendency to focus on physical symptoms. Junior staff were often unfamiliar with tools intended for measuring quality of life. To address these gaps, several groups proposed targeted training programmes and routine use of standardised assessment tools. Cascade training was identified as a feasible solution for capacity building. These findings mirror international studies showing that training gaps contribute to hesitancy in adopting a palliative approach, especially in respiratory and rehabilitation settings [10,11]. In Malaysia, these challenges are compounded by the relatively recent recognition of palliative care as a specialty and its limited inclusion in medical curricula [13].

Resource constraints, such as limited time, consultation space, and spirometry equipment, further hindered comprehensive assessments. Patient fatigue and mobility limitations also restricted attendance. Suggested solutions included redesigning clinic workflows, involving caregivers, and advocating for greater resource allocation. These practical barriers are commonly reported in low-and middle-income countries (LMICs) [14] and must be addressed through system-level investment and planning.

System-level barriers included under-implemented programmes, fragmented referrals, and poor continuity of care. Participants suggested strengthening shared care protocols, creating priority lanes for severe COPD patients, and revitalising outreach services. These strategies align with international calls for greater coordination between primary and secondary services to improve care integration [15].

### Managing severe COPD: practical challenges and infrastructure gaps

Participants outlined significant challenges in the management of severe COPD, especially in relation to pulmonary rehabilitation, palliative care integration, and systemic constraints. Pulmonary rehabilitation, for example, was viewed as both underutilised and inaccessible in Malaysia, largely due to limited availability of services, insufficient resources, and the absence of clear referral pathways within the public healthcare system. Few physiotherapists had received formal training, and services were often centralised in urban hospitals. Suggested actions included decentralising pulmonary rehabilitation (*e.g.* *via* telerehabilitation) and fostering interprofessional collaboration. These strategies are consistent with global evidence that recommend adopting pulmonary rehabilitation models for community-based delivery, particularly in LMICs.

Palliative care integration was rarely achieved. Most participants had not initiated palliative referrals for severe COPD patients, citing uncertainty about timing, lack of confidence, and difficulty accessing opioids. Concerns about regulatory restrictions were also raised. Recommendations included creating more explicit referral criteria, expanding professional training, and engaging policymakers to ensure appropriate opioid access. These challenges echo global findings from resource-constrained contexts, where palliative services are more often linked to oncology than to chronic illnesses such as COPD [16].

Patient-level barriers, such as low disease awareness, late presentation, cultural beliefs, and emotional responses (*e.g.* guilt, fatalism) further complicated care delivery. Participants recommended strengthening health education and communication skills among HCPs, while also improving clinic environments to support private, empathetic discussions. These issues highlight the importance of culturally sensitive engagement strategies that prioritise patient understanding and trust.

Systemic issues included the limited education and training of HCPs in non-pharmacological and supportive care approaches, contributing to a predominant reliance on pharmacological management. Additionally, the lack of integrated, community-based palliative services further constrained comprehensive care delivery. High out-of-pocket costs, travel burdens, and long wait times discouraged continuity of care. Suggested reforms included decentralised care delivery, better inter-sectoral collaboration, and greater support for non-pharmacological interventions. These structural limitations are widely recognised in LMICs and require both policy and health systems reform to enable more responsive care models.

### Cascade training and community models: implementation potential

Cascade training emerged as a key recommendation across multiple groups. Participants believed it could improve knowledge dissemination, communication skills, and care coordination. International evidence supports this approach, particularly in resource-limited settings where cascade models have been used effectively to upskill frontline providers with limited access to formal training [10,11].

In parallel, participants recognised the difficulty in identifying severe COPD patients suitable for palliative care due to unpredictable disease trajectories. Tools such as the Supportive and Palliative Care Indicators Tool and the Body Mass Index, Obstruction, Dyspnoea, and Exercise Capacity Index were discussed as potential supports, although awareness and use remain limited [8,17]. These challenges were consistent with international findings, where prognostic uncertainty and the lack of disease-specific guidance hindered timely palliative care integration, particularly in comparison to cancer [17].

There was a clear call for national guidelines to support early palliative integration. However, participants noted that guidelines must be accompanied by actionable referral pathways, institutional support, and implementation strategies tailored to local realities [16]. Models such as nurse-led coordination, successfully implemented in the UK, Canada, and Thailand, offer practical examples for adaptation in Malaysia [12]. Evidence from a population-level study in Belgium indicates that early palliative home care for patients with COPD was associated with significantly reduced hospitalisations and lower healthcare costs, along with increased uptake of general practitioner services and home-based care [18]. These findings support the potential value of early, community-based palliative integration in improving outcomes and system efficiency.

Finally, participants highlighted the urgency of engaging policymakers to improve opioid access and support decentralised models of care. These systemic issues remain significant barriers to effective palliative integration in Malaysia and were echoed in global literature [16].

### Participant reflections: enriching perspectives on workshop experience

An anonymous post-workshop feedback survey, completed by all 25 participants, indicated that the workshop successfully met its objectives, with 100% agreement on objective achievement and 92.9% expressing satisfaction with the overall outcomes. To incorporate participant voices, we integrated anonymised written responses from this feedback survey. These reflections offer qualitative insights into the workshop’s value and provide direction for future improvements.

One participant called for broader professional inclusion, stating, ‘Can include general practitioners (private), they can share and highlight the problems they face in managing patients with end-stage COPD’, and further added, ‘Can include paramedics, for example, COPD nurse educators’ (R1), underscoring the perceived need for a more multidisciplinary perspective.

The group-based learning format was well received. Out of 25 participants, five explicitly highlighted the small group discussions or interdisciplinary sharing as the most useful aspects of the workshop.

Participants also offered feedback on the workshop structure and content. One described it as ‘Very good overall’ (R4), while another recommended ‘More small group discussion and basic pulmonary rehab training’ (R9). Several expressed interest in continued engagement, with comments such as ‘Repeat it’ (R5) and ‘Similar workshops would be useful in the future’ (R18).

These reflections highlighted the perceived value of the workshop’s interactive, multidisciplinary format and provided clear suggestions for enhancing future iterations. However, the workshop was conducted with a relatively small group of HCPs (n = 25), and findings may not be generalisable beyond the specific context of the Malaysian public healthcare system. The discussion-based format, while valuable for eliciting insights, did not employ formal consensus or voting methods, and responses may have been influenced by group dynamics or participant bias. These limitations should be considered when interpreting the applicability of the proposed solutions.

## CONCLUSIONS

This workshop provided a valuable platform for multilevel stakeholders involved in palliative care and severe COPD to share challenges and collaboratively propose practical solutions. The discussions underscored that patients with severe COPD often experience unrecognised and unmet palliative care needs, particularly within outpatient and primary care settings. Moving forward, it is essential to actively involve patients and caregivers in designing care pathways to ensure that services are responsive to lived experiences and preferences. Rigorous evaluation of the proposed strategies will be critical to guide sustainable health system reform and inform national policy development.
